# Standalone Axial Malrotation after Pediatric Supracondylar Fracture Does Not Seem to Be an Indication for Immediate Postoperative Revision Surgery

**DOI:** 10.3390/children9071013

**Published:** 2022-07-08

**Authors:** Frederik Greve, Michael Müller, Markus Wurm, Peter Biberthaler, Georg Singer, Holger Till, Helmut Wegmann

**Affiliations:** 1 Department of Trauma Surgery, Klinikum rechts der Isar, Technical University of Munich, 81675 Munich, Germany; michael.mueller@mri.tum.de (M.M.); markus.wurm@mri.tum.de (M.W.); peter.biberthaler@mri.tum.de (P.B.); helmut.wegmann@mri.tum.de (H.W.); 2 Department of Pediatric and Adolescent Surgery, Medical University of Graz, 8063 Graz, Austria; georg.singer@medunigraz.at (G.S.); holger.till@medunigraz.at (H.T.)

**Keywords:** pediatric supracondylar humeral fracture, axial malrotation, rotational spur, long-term outcome

## Abstract

Rotational spurs as evidence for post-surgical malrotation are frequently observed when treating pediatric supracondylar humeral fractures (SCHFs). This study aimed to investigate the long-term outcome of a pediatric cohort with unrevised axial malrotation and to discuss the indication for revision surgery. Postoperative radiographs of children treated for SCHFs over eight years were retrospectively analyzed. Children with radiological signs of malrotation (von Laer malrotation quotient) were invited for a follow-up clinical and radiological examination. Among 338 treated children, 39 (11.5%) with a mean age of 5.3 years (range 1.8–11.7 years) showed radiological signs for postoperative malrotation and were not revised and therefore invited to participate in the study. Twelve patients (31%) with a mean age of 11.3 years (range 8.8–13.8 years) took part in the follow-up examination after a mean of 7.1 years (range 5.4 to 11.3 years). The mean postoperative van Laer malrotation quotient was 0.15 (range 0.11–0.2). At follow-up, the range of motion of the elbow joint was not significantly different compared to the contralateral side. Apart from the humeral ulnar angle (*p* = 0.023), there were no significant differences in the radiological axes. The Flynn criteria were excellent and good in 90% of the cases. The mean was 1.7 points indicating excellent subjective results. Standalone postoperative malrotation did not lead to an adverse long-term outcome in a small cohort of pediatric patients with SCHFs and did not indicate immediate postoperative revision surgery. However, further investigations with larger cohorts should verify whether additional criteria such as stability of the osteosynthesis and signs for increasing valgus or varus displacement in the follow-up radiographs should get more importance in decision making.

## 1. Introduction

With a reported prevalence of 12–17% of all pediatric fractures, supracondylar humeral fractures (SCHF) are the most common elbow fractures in children [1,2,3]. Typical trauma mechanisms are falls on the outstretched, non-dominant hand resulting in extension fractures (98%) due to hyperextension of the elbow. Less frequently, flexion fractures (2%) occur due to direct trauma to the olecranon with the arm in a flexed position [4].

**Figure 1:**
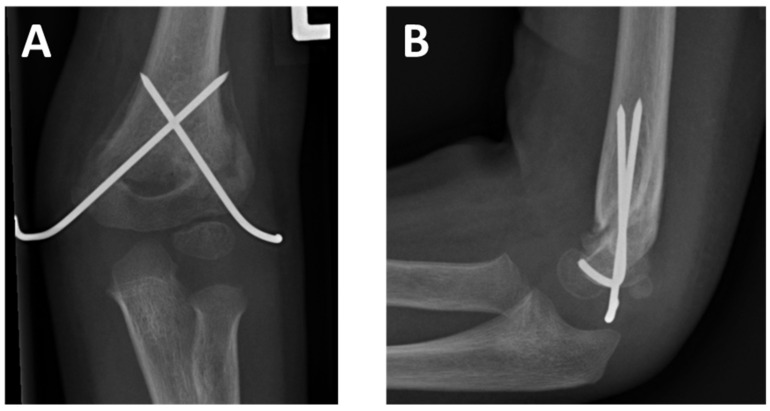
Radiograph of a supracondylar humeral fracture treated by percutaneous crossed pinning in anterior-posterior (**A**) and lateral view (**B**).

**Figure 2:**
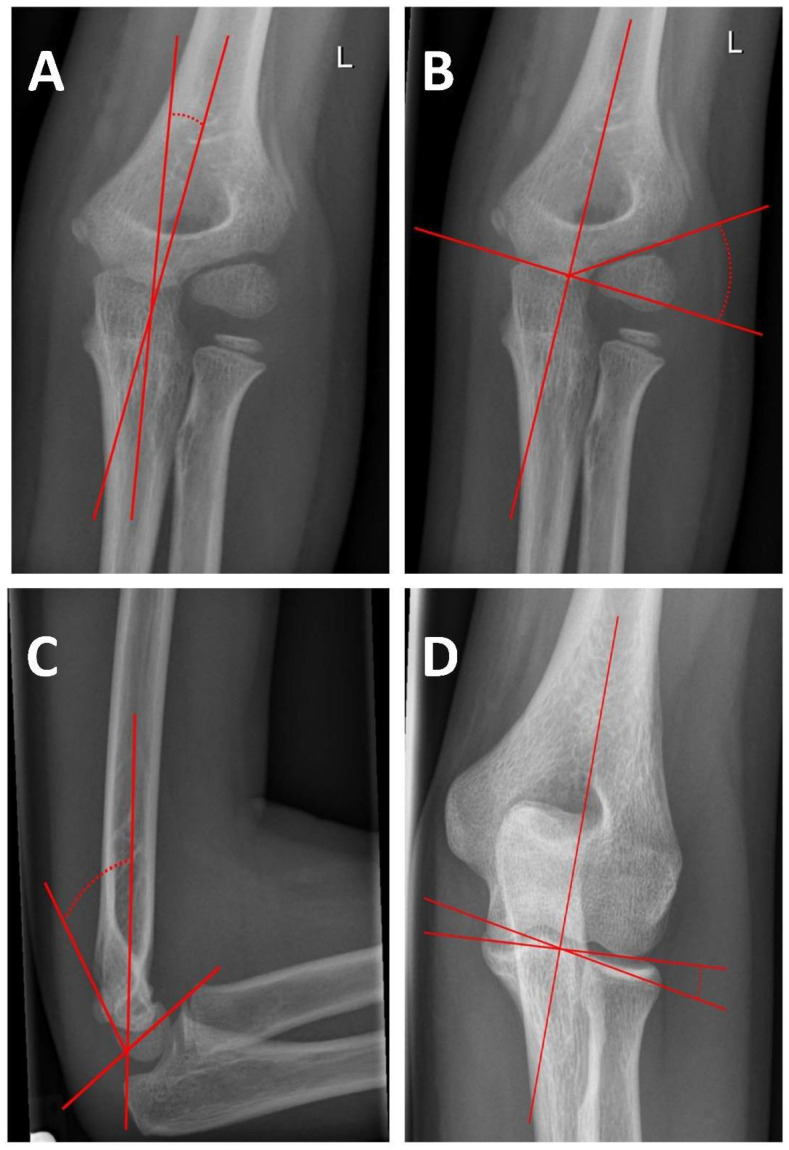
Angles for evaluation of reduction in supracondylar humeral fractures. (**A**): Humeral ulnar angle. An increased angle indicates elbow valgization and a decreased angle elbow varization. (**B**): Baumann’s angle. An increased angle indicates humeral varization and a reduced angle humeral valgization. (**C**): Antecurvation angle. A decreased angle indicates antcurvation and an increased angle recurvation. (**D**): Humerus trochlear angle. An increased angle indicates elbow valgization and a reduced angle elbow varization.

Treatment of choice of displaced SCHFs consists of closed reduction and osteosynthesis either by percutaneous lateral pinning, percutaneous crossed pinning (PCP), or antegrade nailing (AN). After fixation, radiological imaging is essential (Figure 1). Baumann’s angle and the humeral trochlea angle help to determine the correct reduction in the frontal plane, whereas the antecurvation angle and the anterior humeral line assess the sagittal plane (Figure 2) [4,5,6]. Spontaneous correction of minor malreduction in the sagittal plane is believed to be possible up to seven years [7]. However, remodeling of frontal plane deviations and rotational malalignment is less reliable. Rotational deformity might lead to subsequent tilting of the distal fragment into the cubitus varus or valgus deformity [8]. Cubitus varus deformity (CVD) is the most frequent complication after SCHF [9,10,11]. Chronic CVD is associated with posterolateral rotational instability, chronic elbow pain, and functional impairment, which might indicate complex revision surgeries in up to 15% of the cases in the long-term [12,13,14].

**Figure 3:**
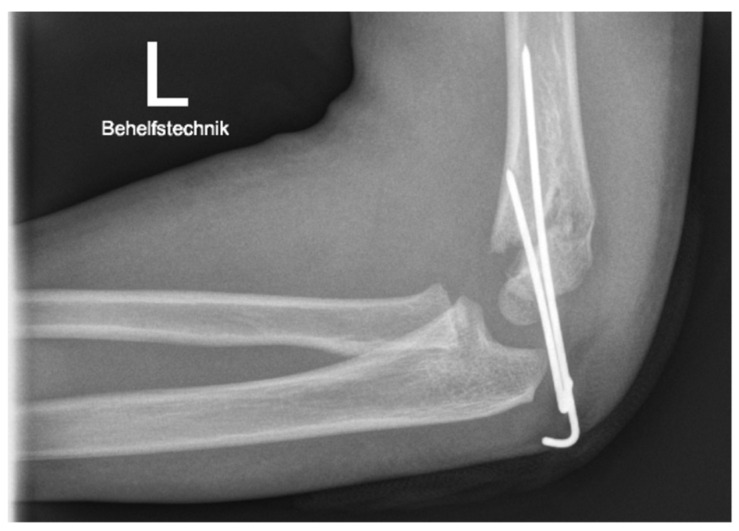
Postoperative radiograph of a rotational spur in the lateral view of a four-year-old patient after Gartland type 3 fracture and treatment by PCP.

Persisting rotational malalignment is a risk factor for the development of CVD. Based on the anatomy of the distal humerus, even minor axial malrotation of the distal fragment relative to the humerus significantly reduces the overlapping area of the fracture fragments, resulting in instability. This might initiate secondary displacement in the frontal plane with increased risk for the development of CVD [14]. Rotational spurs in the lateral radiograph (Figure 3) are a radiological sign of malrotation. While persisting deviations in the sagittal plane are easy to detect, rotational deformities might be missed during surgery [15]. They are visualized in postoperative radiographs and are subject to controversial discussion regarding the indication for revision surgery.

Due to the limited number of reports describing the long-term outcome of persisting malrotation after closed reduction and stabilization of displaced SCHFs, this study aimed to investigate the long-term effect of a pediatric cohort with unrevised axial malrotation and to discuss the indication for revision surgery.

## 2. Materials and Methods

This is an explorative single-center cohort study investigating the radiological and functional outcome of children with unrevised axial malrotation after surgical treatment of displaced SCHFs.

### 2.1. Patient Collective

Post-operative radiographs of pediatric patients who underwent closed reduction and percutaneous or intramedullary fixation of displaced SCHFs over eight years were analyzed retrospectively. If persisting malrotation was detected, the children were included in this study and invited for a long-term follow-up examination. Exclusion criteria were open surgical treatment, additional fractures of the forearm, and a positive history of fractures of the ipsilateral and contralateral elbow.

### 2.2. Retrospective Analysis

The patients’ medical data were analyzed for general information (age, sex, affected side), Gartland classification, fracture morphology (oblique or transverse fracture), and surgery performed.

**Figure 4:**
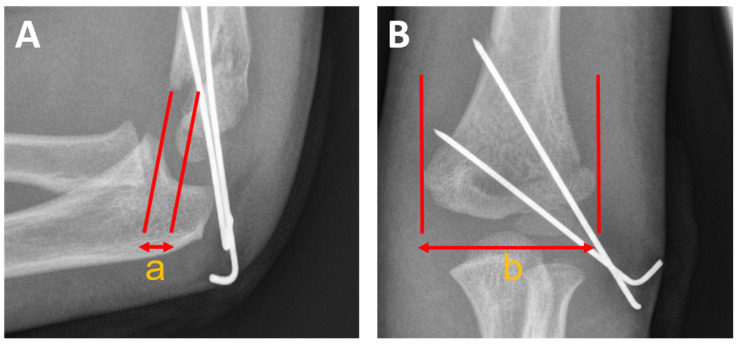
Calculation of the von Laer malrotation quotient (rfq). (**A**): Dimension of the rotational spur in millimeters ‘a’. (**B**): Dimension of the distal fragment ‘b’ in the anterior-posterior plane. For calculating the von Laer malrotation quotient (rfq), ‘a’ is divided by ‘b’ (a/b).

Malrotation was quantified by applying the von Laer malrotation quotient (rfq) [14]. The dimension of the malrotated displaced humeral metaphysis (rotational spur) in the sagittal plane in millimeters is divided by the size of the distal fracture fragment in the antero-posterior plane (Figure 4). Two experienced investigators performed measurements in agreement.

### 2.3. Follow-Up Examination

#### 2.3.1. Clinical Examination

Patients presenting for the follow-up examination were analyzed for the range of motion (flexion/extension, supination/pronation) of the affected and contralateral elbow joint by applying an inclinometer (Baseline^®^ Wrist Inclinometer, Fabrication Enterprises, Inc., Elmsford, NY, USA). The range of motion was measured three times by a single investigator. The results are presented as the average of the measurements. The Yamamoto angle was determined [16] to assess the internal rotation deformity as part of CVD. Therefore, the patient was asked to perform a maximal internal rotation of the shoulder with the elbow at 90° flexion on the back and the shoulder held at the maximum extension with the upper body in flexion. The angle was measured between a horizontal line and the long axis of the forearm held in maximal internal rotation. A positive angle corresponds to an increase in internal rotation, and a negative angle corresponds to a decrease in internal rotation. Patients with CVD have an increased degree of possible internal rotation at the affected side compared to the unaffected side. The measured angles of the formerly injured side were compared to the contralateral side.

#### 2.3.2. Radiological Examination

Standard radiographs of the affected and unaffected elbow in the anterior-posterior and sagittal plane were performed. The following angles were measured in the agreement of two experienced investigators.

##### Humeral Ulnar Angle

The humeral ulnar angle is formed in the anterior-posterior plane by the ulnar length axis and the humeral length axis when the arm is supinated and extended (Figure 2A).

##### Baumann’s Angle

Baumann’s angle was assessed in an antero-posterior radiograph. There are deviating definitions for Baumann’s angle in the literature [5]. In this work, the angle is defined by the angle between a line parallel to the lateral condylar physis and a perpendicular line to the axis of the humeral shaft (Figure 2B).

##### Antecurvation Angle

This angle was measured in the sagittal plane. It is formed by the humeral length axis and a vertical line to the axis of the epiphyseal plate (Figure 2C). In the case of already closed epiphyseal plates, the angle is formed by the ventral tangent of the trochlea and the humeral length axis.

##### Humeral Trochlear Angle

The humeral trochlea angle is formed in the antero-posterior plane by a tangent of the distal joint surface of the trochlea and the length axis of the humerus and is used in the case of closed epiphyseal plates (Figure 2D).

#### 2.3.3. Scores

Patients were asked to complete the “quick Disability of the Arm, Shoulder and Hand Outcome Measure” (QuickDASH) test. A value of 0 accounts for the best possible test result and indicates the least impairment in daily life. Values up to 20 points account for very good subjective results [17].

For the comparison of objective results, the Flynn criteria were assessed. Here, the effect is evaluated based on the loss of range of motion in the elbow joint and the change in the cubital axis. The worse criterion in each case is decisive [18].

### 2.4. Statistics

Descriptive analysis and statistical testing were performed with GraphPad Prism Version 9.2.0 (San Diego, CA, USA). Normal distribution was tested with the D’Agostino Pearson test. For comparison of paired groups, the paired *t*-test was used in the case of normal distribution, and the Wilcoxon test was used in the case of non-normally distributed values. A comparison of the rfq between the follow-up patients and the whole study population was performed with the Mann–Whitney *U* test. The level of significance was set as *p* < 0.05.

### 2.5. Ethics

Before the study, all patients and/or their legal representatives obtained informed consent. Ethical approval was obtained from the local Ethics Committee of the Medical University of Graz, Austria (29-094 ex 16/17, date of acceptance: 30 January 2018).

## 3. Results

### 3.1. Retrospective Analysis

During eight years, 338 children were operatively treated for displaced SCHFs. Of these, 39 children (11.5%) with a mean age of 5.3 years (range 1.8–11.7 years) with signs of persisting malrotation in the postoperative radiograph were identified.

A pre-surgical radiograph was available in 34 cases. Six patients (17%) suffered from a Gartland type 2 fracture and 28 (83%) from a Gartland type 3 fracture.

An allocation of the fracture type (oblique vs. transverse) was possible in 35 cases. Patients were either treated with PCP or AN (Table 1).

All children were immobilized in an upper arm cast for an average of 30 days (range 19–45 days) in the PCP group and average of 23 days (range 11–32 days) in the AN group, respectively. Implant removal coincided with cast removal in the PCP group. In the AN group, implant removal was performed after a mean of 116 days (range 22 to 249 days).

The malrotation was quantified using the von Laer malrotation quotient (rfq), calculated from post-surgical radiographs (Figure 4). The mean rfq was 0.18 (range 0.11–0.41).

### 3.2. Follow-Up Examination

Twelve of the 39 patients (31%) were presented for a follow-up examination. The mean age at the time of injury was 4.2 years (range 1.8–9 years). The mean age at the follow-up examination was 11.3 years (range 8.8–13.8 years), and the mean follow-up time was 7.1 years (range 5.4–11.3 years). Details of the follow-up study population are depicted in Table 2.

Patients treated with PCP underwent immobilization for 19, 24, and 45 days, respectively. Among the nine individuals treated with AN, three patients received additional immobilization for 23, 27, and 32 days, and the mean time until implant removal was 142 days (range 39–240 days). The mean rfq in the follow-up group was 0.15 (range 0.11–0.2) and was not significantly different from the whole study population (*p* = 0.784). 

#### 3.2.1. Range of Motion

The range of motion was compared to the contralateral, healthy elbow. Values for flexion, extension, pronation, supination, and Yamamoto angle as an approximation for internal rotation are depicted in Table 3 and were not significantly different comparing the formerly injured to the contralateral elbow.

#### 3.2.2. Radiological Analysis

Humeral ulnar angle, antecurvation angle, humeral trochlear angle, and Baumann’s angle were analyzed. Comparing the formerly injured to the unaffected side, there was a significant (*p* = 0.023) increase in the humeral ulnar angle (valgization). We detected minor increases in the antecurvation and humeral trochlea angle (varization). Baumann’s angle was slightly decreased (varization). There was no radiological hint for malalignment or CVD. Details are depicted in Table 4.

#### 3.2.3. Scores

The Flynn criteria showed excellent results in six patients (50%), good results in five patients (40%), and satisfactory results in one patient (10%). None of the patients presented poor results.

The QuickDASH score showed similar results with a mean of 1.7/100 points (range 0–6.8 points).

## 4. Discussion

The study’s most important finding was that persisting standalone malrotation after surgical fixation of a pediatric supracondylar humeral fracture did not lead to an adverse radiological outcome or functional impairment in a long-term follow-up of 12 pediatric patients over seven years. According to our results, immediate postoperative revision surgery does not necessarily seem to be indicated in the case of standalone axial malrotation.

Displaced SCHFs are treated with closed reduction followed by fixation after intraoperative fluoroscopic verification of anatomical alignment. Baumann’s angle and the humeral ulnar angle may help to evaluate the proper reduction in the frontal plane. In contrast, the tilt of the humeral capitulum and anterior humeral line serves for reduction evaluation in the sagittal plane.

Despite the best possible effort, reduction can be unsatisfying in postoperative radiological control. A well-known sign of rotational malreduction is the presence of a rotational spur, as depicted in Figure 1. Despite comprehensive research for almost a century, the literature regarding guidance in the presence of rotational spur is limited.

With several underlying risk factors (e.g., obesity and fracture displacement), malreduction is a frequent problem in treating SCHFs [19,20]. We detected a rotational spur in 11.5% of our cases. Compared to studies with a reported incidence of 98% in 1978 [14], 23.3% in 2015 [19], and 5.6% in 2020 [20], radiological outcomes improved over the years. This might be associated with a better understanding of rotationally stable reduction techniques. Today, osteosynthesis using k-wire fixation in varying configurations of pin placement is the gold standard [6,21,22,23,24]. In our collective, a rotational spur was present after PCP and AN osteosynthesis. In younger children, deviations in the sagittal plane such as increased antecurvation usually remodel without further intervention. However, malrotation is not supposed to remodel [7,8]. Axial malrotation might cause tilting of the distal fragment in the frontal plane due to a decreased contact area between the proximal and distal portions, leading to severe CVD, which is the most frequent complication after SCHFs [14]. 

To understand CVD following SCHF, it is essential to distinguish between the divergent biomechanical characteristics of oblique and horizontal fracture patterns. In oblique fractures, rotational shearing of the fracture fragments directly leads to varus malalignment of the distal fragment. In horizontal fractures, axial malrotation is followed by decreased fragment contact, which might initiate a collapse of the medial epicondyle, subsequently leading to varization of the cubital axis. Thus, in contrast to oblique fractures, axial rotation in transverse fractures without tilting the distal fragment does not necessarily lead to CVD.

The rotational spur might initially cause a deficit of flexion. Due to fast resorption and proximal migration of the spur during further growth, this impairment exists only temporarily. 

Suppose a rotational spur is detected in the postoperative radiograph. In that case, surgeons must choose between revision surgery, with possible associated complications, to prevent future CVD development, or a watch-and-wait strategy. For the prediction of the development of CVD, it is essential to evaluate whether tilting of the distal fragment yet exists or progresses and subsequently might lead to cubitus varus or if the performed osteosynthesis provides sufficient stability to the fracture and prevents further tilting.

Among several existing options [25,26,27,28,29,30], the rfq developed by van Laer et al. was used to determine postoperative rotational malalignment [14] in this study. However, the rfq and the size of the rotational spur do not correlate with the exact clinical rotational malalignment [14,31]. More precise methods using computer tomography for rotational analysis are available but are hardly conductible in ethical terms due to radiation exposure. The mean calculated rfq of 0.18 in the entire study population and 0.15 in the follow-up group is comparable to 0.16 in the introduction study by von Laer et al. [14]. In their collective, revision surgery was considered in 15% of the cases due to subjective cosmetic and social issues—none of the patients in our collective aimed for revision surgery.

In our cohort, in contrast to the expected varus deformation, the radiological analysis revealed a significant valgization of the humeral ulnar angle but no significant differences between the trochlea and Baumann’s angle. In the sagittal plane, the tilt of the humeral capitulum was almost identical to the unaffected side.

Regarding the range of motion, there was a slightly decreased flexion and pronation and a minor increase in extension on the injured arm. There was no difference in supination between the injured and contralateral sides. Analysis of the Yamamoto angle excluded internal rotation deformity.

When CVD is defined as the triad of varus, hyperextension, and internal rotation deformity [32], none of the patients was diagnosed with CVD during follow-up examination.

With 90% excellent and good results of the Flynn criteria and very good results of the QuickDASH score, there were no poor results in the follow-up examination group.

The results of this study allow for toleration of axial malrotation in postoperative X-ray controls, as there is no need to fear long-term functional impairment. However, this requires stable osteosynthesis with no malalignment in the frontal plane. It is crucial to rule out the secondary displacement of the distal fragment, tilting in a varus position due to the decreased contact area of the fragments following rotational malalignment in the presence of an unstable fixation. The nature of transverse and oblique fracture morphology must be understood and appreciated when the decision for revision surgery is made.

It is important to note that this study is not without limitations. The homogeneity of our patient collective is limited due to vast age distribution and varying surgical procedures (AN and PCP). Our follow-up rate of 31% is low but still acceptable. However, a more extensive study population would have benefited our interpretation of definitive treatment recommendations. Therefore, our results have to be interpreted with caution. Data was allocated with the best possible care. Multiple testing (including numerous investigators) at deviating timepoints for test-retest reliability assessment would have benefited our results’ accuracy. Another limitation is the retrospective design of this study. Subjective concerns regarding the individual outcome might have driven patients to present for a follow-up examination. This is a well-known bias of retrospective studies with follow-up invitations. Hence, prospective studies with a more extensive population must reinforce our derived statement.

## 5. Conclusions

Stable fixed malrotation following surgical treatment of SCHFs revealed slight radiological signs of valgization. Still, it did not lead to adverse subjective or functional outcomes in a small cohort of 12 pediatric patients in long-term follow-up. As a clinical consequence of our results, we advocate that revision surgery is not necessarily indicated in the case of standalone malrotation (no tilting in varus or valgus) in young patients. However, in case of signs of postoperative malrotation, close follow-up is crucial to detect ongoing tilting as an indication for revision surgery and prevent future CVD with its long-term sequelae such as cosmetic issues, ulnar nerve dysfunction, and medial instability and functional impairment [33,34,35,36,37].

## Figures and Tables

**Table 1:** The study population of 39 patients with supracondylar humerus fracture and postoperative malrotation.

**Sex**	19 (49%) male	20 (51%) female
**Affected side**	11 (28%) right side	28 (72%) left side
**Fracture geometry**	19 (54%) transverse	16 (46%) oblique
**Gartland classification**	6 (17%) type 2	28 (83%) type 3
**Surgery**	14 (36%) PCP	25 (64%) AN

**Table 2:** The study population of 12 patients with postoperative malrotation who underwent a follow-up examination.

**Sex**	7 (58%) male	5 (42%) female
**Affected side**	3 (25%) right side	9 (75%) left side
**Fracture geometry**	7 (58%) transverse	5 (42%) oblique
**Gartland classification**	4 (33%) type 2	8 (67%) type 3
**Surgery**	3 (25%) PCP	9 (75%) AN

**Table 3:** The assessment of the range of motion and Yamamoto angle as measurements for internal rotation.

	Injured Side Mean/Median (Range)	Contralateral Side Mean/Median (Range)	Difference Mean/Median (Range)	*p*-Value
**Flexion**	143°/143° (130°–150°)	145°/145° (135°–150°)	1°/0° (−12°–5°)	0.268 #
**Extension**	18°/15° (10°–25°)	15°/13° (10°–20°)	3°/0° (−5°–20°)	0.266 §
**Pronation**	84°/85° (60°–100°)	88°/90° (60°–100°)	4°/3° (−20°–10°)	0.176 §
**Supination**	103°/98 (70°–115°)	103°/100° (80°–115°)	0°/0° (−15°–20°)	>0.999 #
**Yamamoto**	−2° (–30°–40°)	−4° (−30°–20°)	2°/0° (−20°–20°)	0.516 #

#: paired *t*-test; §: Wilcoxon test.

**Table 4:** The radiological analyses specific to SCHFs (* = statistically significant difference).

	Injured Mean/Median (Range)	Unaffected Mean/Median (Range)	Difference Mean/Median (Range)	*p*-Value
**Humeral ulnar angle**	7°/8° (2°–11°)	11°/10 (8°–18°)	3°/3° (−1°–9°)	0.023 * §
**Antecurvation angle**	50°/45° (37°–76°)	50°/51° (31°–73°)	0.7°/1° (−27°–16°)	0.847 #
**Baumann’s angle**	70°/70° (60°–83°)	67°/74° (46°–79°)	3°/5° (−14°–9°)	0.688 §
**Humerus trochlear angle**	2°/2° (−5°–7°)	5°/4° (2°–10°)	3°/1.5° (−3–8°)	0.313 §

#: paired *t*-test; §: Wilcoxon test.

## Data Availability

The data used in this study can be obtained from the corresponding author in case of reasonable request.
